# Low Working Temperature of ZnO-MoS_2_ Nanocomposites for Delaying Aging with Good Acetylene Gas-Sensing Properties

**DOI:** 10.3390/nano10101902

**Published:** 2020-09-23

**Authors:** Sijie Wang, Weigen Chen, Jian Li, Zihao Song, He Zhang, Wen Zeng

**Affiliations:** 1 State Key Laboratory of Power Transmission Equipment & System Security and New Technology, Chongqing University, Chongqing 400044, China; 201911021046@cqu.edu.cn (S.W.); lijian@cqu.edu.cn (J.L.); 201711131056@cqu.edu.cn (Z.S.); 2 School of Electrical Engineering, Chongqing University, Chongqing 400044, China; 3 School of Electrical Engineering, Zhengzhou University, Zhengzhou 450001, China; zhanghe@zzu.edu.cn; 4 School of Materials Science and Engineering, Chongqing University, Chongqing 400044, China

**Keywords:** pure MoS_2_, ZnO-MoS_2_ nanocomposites, gas sensors, aging, gas-sensing mechanism

## Abstract

The long-term stability and the extension of the use time of gas sensors are one of the current concerns. Lowering the working temperature is one of the most effective methods to delay aging. In this paper, pure MoS_2_ and ZnO-MoS_2_ nanocomposites were successfully prepared by the hydrothermal method, and the morphological characteristics were featured by scanning electron microscopy (SEM), X-ray diffraction (XRD), transmission electron microscopy (TEM) and X-ray photoelectron spectroscopy (XPS). Pure MoS_2_ and ZnO-MoS_2_ nanocomposites, as a comparison, were used to study the aging characteristic. The sensing properties of the fabricated gas sensors with an optimal molar ratio ZnO-MoS_2_ (Zn:Mo = 1:2) were recorded, and the results exhibit a high gas-sensing response and good repeatability to the acetylene detection. The working temperature was significantly lower than for pure MoS_2_. After aging for 40 days, all the gas-sensing response was relatively attenuated, and pure MoS_2_ exhibits a faster decay rate and lower gas-sensing response than nanocomposites. The better gas-sensing characteristic of nanocomposites after aging was possibly attributed to the active interaction between ZnO and MoS_2_.

## 1. Introduction

In a high-voltage power system, power transformers mainly work for electricity generation and transmission with high expense [1]. It is critical to ensure the safety and reliability of power transformers during the operation for industrial production [2,3]. At present, oil-immersed power transformers are mainly popular in China [4,5]. Hydrocarbons in the insulating oil will decompose following thermal or electrical failure, causing the C–H bond and C–C bond breaking and forming gas molecules [6,7,8,9]. Therefore, dissolved gas analysis (DGA), as a diagnostic method for potential faults, is effective in oil-immersed power transformers. Meanwhile, the optimal operating temperature of gas sensors is usually 200–500 °C, which will not only cause high-energy power consumption but will also cause the growth of oxide grains in long-term use. This is the major factor causing long-term stability [10,11,12,13]. Matsuura et al. [14] found that SnO_2_ operating at 550 °C for 20 days can cause its grains sizes growing from 22.1 nm to 23.5 nm. Korotcenkov et al. [15] hold the opinion that the change in grain size will change crystal planes, which will affect the conductivity of the gas-sensing layer and the adsorption and desorption rate of reducing gas on the surface of gas-sensing materials. Thus, it is urgent to fabricate a gas sensor with low-energy consumption, low working temperature and long-term stability [16,17,18,19].

In the past few decades, many investigations have pointed out that molybdenum sulfide (MoS_2_) and other two-dimensional nanomaterials are developing rapidly, stably and controllably because of its unique properties with widely applying in optical and electronic devices, especially in gas sensors [20,21,22,23]. Therefore, MoS_2_, as a narrow-band-gap material, is beneficial to the development of low-energy-consumption gas sensors [24]. Recently, it has been reported that MoS_2_ can improved the gas-sensing performances via compounding with metal oxide semiconductors (MOSs) such as MoO_3_, ZnO, and SnO_2_. Meanwhile, zinc oxide (ZnO), as a typical n-type MOSs with a direct wide band gap (Eg ≈ 3.37 eV) and exciton binding energy (~60 meV), has superior sensing properties, due to its high mobility of carriers, good chemical and thermal stability [25,26]. Wu et al. [27] successfully prepared ZnO-MoS_2_ n-p heterojunction nanostructures with an extremely high gas-sensing response of 3050%, and the detection limit is 5 μL/L to 50 μL/L H_2_. Kumar et al. [28] proposed the use of MoS_2_-MoO_3_ n-n homojunction micronanoflowers for the effective detection of ethanol gas at room temperature, which achieved a good response to 10~100 μL/L ethanol under low-energy consumption.

Nowadays, stability is a key parameter in the long-term development of gas sensors. The stability of the sensor is divided into two types: One is that, when gas sensors are working in operating conditions such as a high temperature and toxic gas, the stability will be reduced. The other is that, when the sensor is in a normal storage state, changes in ambient humidity and temperature fluctuations will also affect the stability of the sensor. External factors can be caused by improving engineering methods, such as drift compensation, selecting the correct gas system components, adding additional filters and temperature stabilization devices, etc., to eliminate sensor stability problems [29]. For internal causes, calcination and annealing after heating treatment can be used to improve its stability [30]. Doping with other MOSs or compounding composite oxide can also increase the stability of gas sensors [19]. Ultraviolet radiation during the gas-sensing test can also improve stability by lowering the operating temperature [31].

In this study, we prepare the pure MoS_2_ and ZnO-MoS_2_ with four different molar ratios by the hydrothermal method and fabricate planar gas sensors. Next, we measure the temperature characteristics, concentration characteristics, and response–recovery characteristics of these gas sensors to C_2_H_2_ gas before and after aging in a dark vacuum environment. Compared with pure MoS_2_, ZnO-MoS_2_ nanocomposites have a better gas-sensing response and can work at a lower working temperature. Hence, the gas sensors fabricated by ZnO-MoS_2_ nanocomposites can greatly improve gas-sensing properties with better long-term stability. In addition, the mechanism that enables nanocomposite materials to reduce the working temperature for gas sensors to delay aging will be explained in detail in the next section.

## 2. Experimental Section

### 2.1. Materials

Sodium molybdate dihydrate (Na_2_MoO_4_·2H_2_O), thiourea (NH_2_CSNH_2_), citric acid monohydrate (C_6_H_8_O_7_·H_2_O), zinc chloride (ZnCl_2_), sodium hydroxide (NaOH), deionized water and absolute ethanol were purchased from Chuandong Chemical Co., Ltd. (Chongqing, China). To ensure the purity for the prepared gas-sensing materials, all the experimental reagents are analytical reagents and the liquid is pure solvent.

### 2.2. Materials Synthesis

**Synthesis of pure MoS_2_.** Firstly, 2 mmol of Na_2_MoO_4_·2H_2_O and 9 mmol of NH_2_CSNH_2_ were dissolved in 70 mL deionized water and stirred for 1 h. Next, 10 mmol of PVP was added into the solution with stirring for 0.5 h to get a clear precursor solution. Subsequently, the precursor solution was transferred in a sealed autoclave with a stainless-steel shell at 200 °C for 21 h in an oven. The black powder was collected after the autoclave cooled to room temperature and washed several times with absolute ethanol and deionized water. Then, put the black powder in a vacuum oven at 60 °C dry for 12 h. Finally, the pure MoS_2_ was obtained.

**Synthesis of ZnO-MoS_2_ Nanocomposites:** Four different molar ratios of ZnO-MoS_2_ nanocomposites were synthesized by the two-step hydrothermal method. Firstly, 1 mmol MoS_2_ prepared in the above steps was dissolved in 40 mL deionized water with constantly stirring for 10 min. Then, the solution was ultra-sonicated for 10 min to ensure even suspension. Next, 0.1, 0.2, 0.5, and 1 mmol ZnCl_2_ were added separately into the above suspension with 2 mmol NaOH. With constantly stirring for 0.5 h, black uniform precursor liquid was collected, and then transferred to Teflon-lined, stainless-steel autoclave at 180 °C for 16 h. The obtained black powder was washed three times with deionized water and absolute ethanol. Then, dry it at 60 °C for 12 h. Finally, the four kinds molar ratios products were labelled as S1 (Zn:Mo = 1:10), S2 (Zn:Mo = 1:5), S3 (Zn:Mo = 1:2), S4 (Zn:Mo = 1:1).

### 2.3. Characterization

The XRD data of samples were analyzed by X-ray diffraction (XRD, Japanese Rigaku D/max-1200 X-ray diffractometer, Tokyo, Japan) with Cu-Kα ray radiation (λ = 0.154 nm) in the range of 5°~70° at a scanning speed of 2°/min. Material micro-morphology data were acquired by using scanning electron microscopy (SEM) with an FEI Nova 400 field emission scanning electron microscope (FE-SEM, FEI, Hillsboro, MI, USA) and using transmission electron microscopy (TEM, ZEISS, LIBRA 200, Jena, Germany). X-ray photoelectron spectroscopy (XPS, ESCALAB 250 Xi, Waltham, MA, USA) with Al-Kα ray radiation source was used to confirm element composition and chemical valence, and the detection step is set to 0.05 eV and the working current is 20 mA. All the data were corrected based on the C 1 s electron binding energy.

### 2.4. Fabrication of Gas Sensor

**Figure 1:**
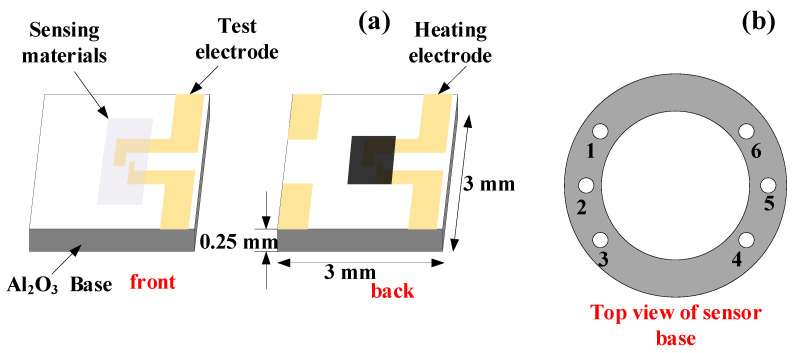
(**a**) Planar sensor substrate construction. (**b**) Sensor base construction.

The fabricated planar sensor is shown in Figure 1, MoS_2_ and ZnO-MoS_2_ were coated on it, respectively, to measure its gas-sensing performance. Firstly, 1 and 6 pins of the sensor base constructions in Figure 1b were soldered with testing electrodes, and 2 and 5 pins were welded with heating electrodes. Next, 20 mg ZnO-MoS_2_ nanocomposites were mixed with absolute ethanol and terpineol with a 2:1 volume ratio in a 5 mL test tube and then sonicated for 10 min to obtain a homogeneous suspension. Subsequently, the coated side of the gas-sensing materials in Figure 1a was evenly coated with suspension liquid. Finally, gas sensors were put in a vacuum oven at 60 °C for 12 h to dry. The planar gas sensors were fabricated successfully after the suspension was dried, and the gas-sensing materials were attached to the substrate with the form of a film.

### 2.5. Gas-Sensing Measurements

**Figure 2:**
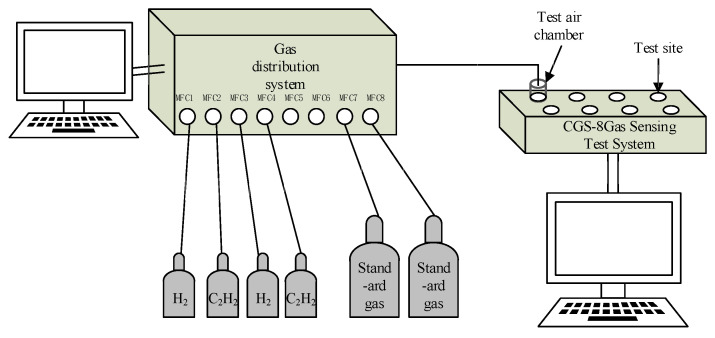
Gas-sensing test flow diagram.

Gas distribution and measurements were included in the gas-sensing process. The RCS 2000-A gas distribution system (Kingsun Electronics Co., Ltd., Beijing, China) contained eight inlet channels and one outlet channel. The basic principle of gas distribution process is to use a sophisticated mass flow controllers (MFCs) to control the gas flow and dilute the target gas to the target concentration with the carrier gas. A CGS-8 TP intelligent gas-sensing analysis system was used to test and analyze gas. The volume of the cylindrical assembled by opaque latex tube air cavity is 100 mL. The flow diagram of the entire gas-sensing test process was shown in Figure 2. The characteristic parameters during the testing process are roughly as follows: resistance in air (R_a_), resistance in reducing gas (R_g_), gas-sensing response (R_a_/R_g_), optimal working temperature, detection limit, response–recovery time.

**Figure 3:**
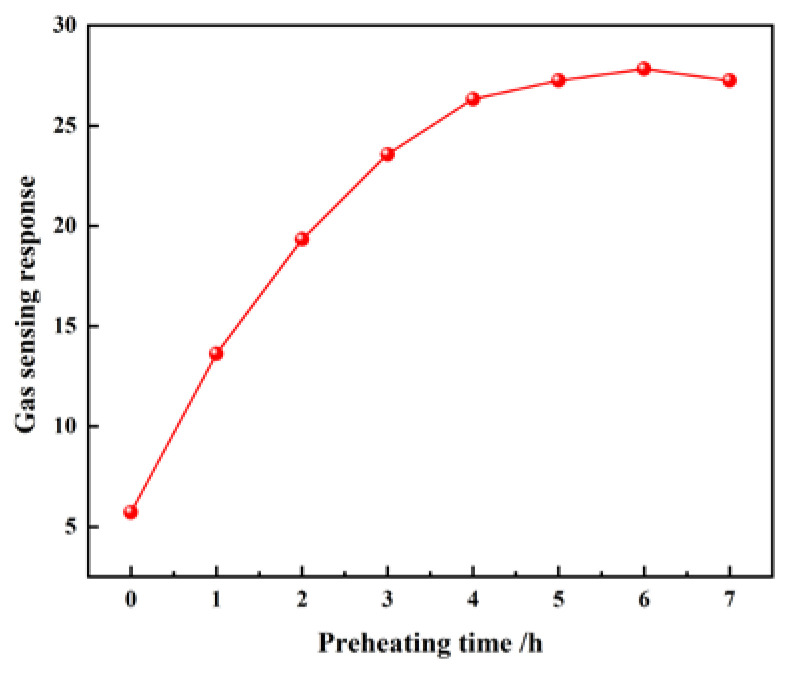
Preprocessing time curve of S3 composite material at 15 μL/L C_2_H_2_.

Before the gas-sensing test, it is necessary to preheat the material to optimize the aging experiment. The preprocessing experiment selected S3 at 15 μL/L of C_2_H_2_, and the results were shown in the Figure 3. The aging experiment was conducted in a dark vacuum environment. The gas-sensing response in this paper was defined as R_a_/R_g_, which R_a_ and R_g_ were the resistances of gas sensors in air and the C_2_H_2_ gas, respectively.

## 3. Results and Discussion

### 3.1. Morphology and Structure

**Figure 4:**
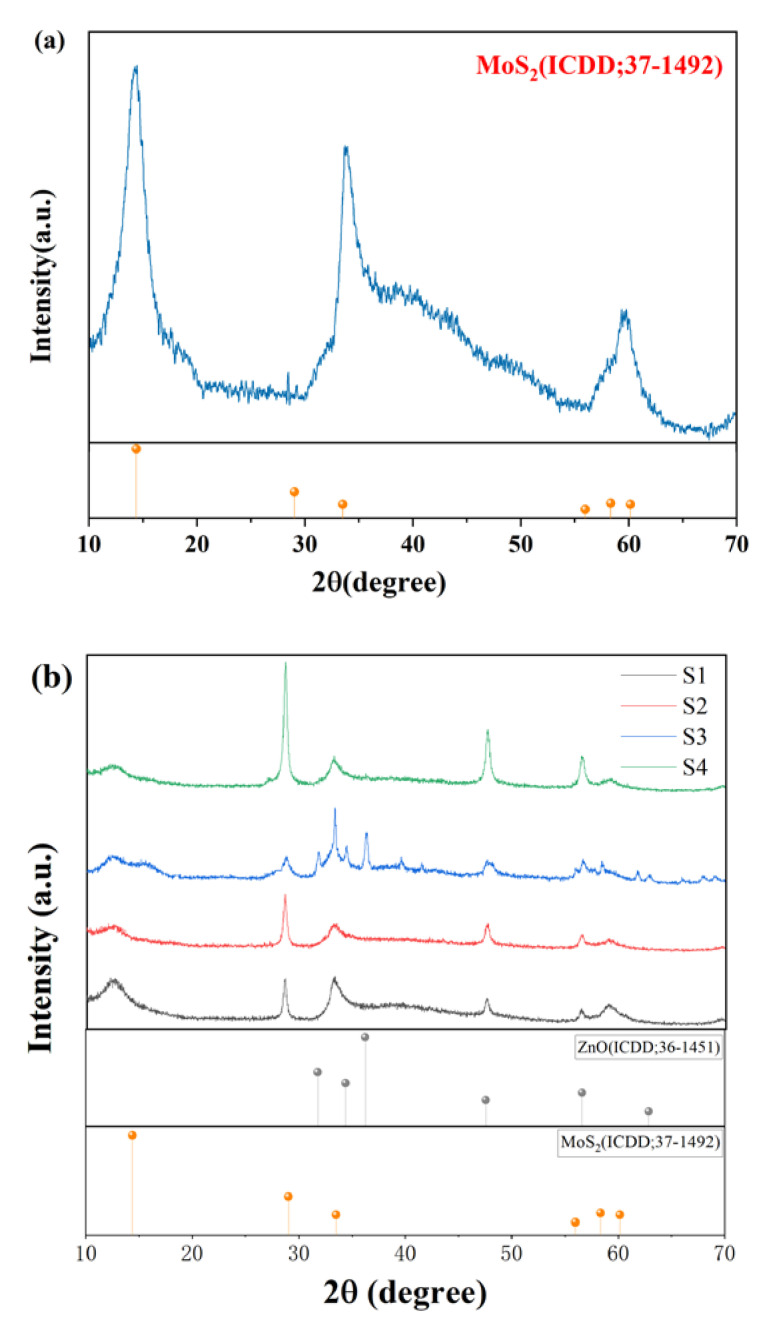
(**a**) X-ray diffraction (XRD) data of MoS_2_ and (**b**) ZnO-MoS_2_ with different composite ratios.

XRD patterns of pure MoS_2_ and four proportions of ZnO-MoS_2_ nanocomposites are shown in Figure 4. It can be seen that all diffraction peaks of ZnO-MoS_2_ nanocomposites remain highly consistent, which indicates the crystal structures of MoS_2_ do not change with the addition of ZnO particles. Meanwhile, the diffraction peaks of ZnO are not very obvious, but still are highly consistent with the standard hexagonal ZnO pattern (JCPDS No. 36-1451). It shows that only ZnO particles are deposited during the compounding process, indicating that no impurities are introduced. In contrast, it can be found that the diffraction peaks of MoS_2_ are more obvious but present a typical amorphous bun shape with poor crystallinity, which is a common problem for two-dimensional materials. The six peaks at 14.38°, 29.03°, 33.51°, 55.98°, 58.33° and 60.15° of MoS_2_ are greatly indexed as the standard MoS_2_ pattern (JCPDS No. 37-1492) with little impurities, indicating the black powders are MoS_2_.

**Figure 5:**
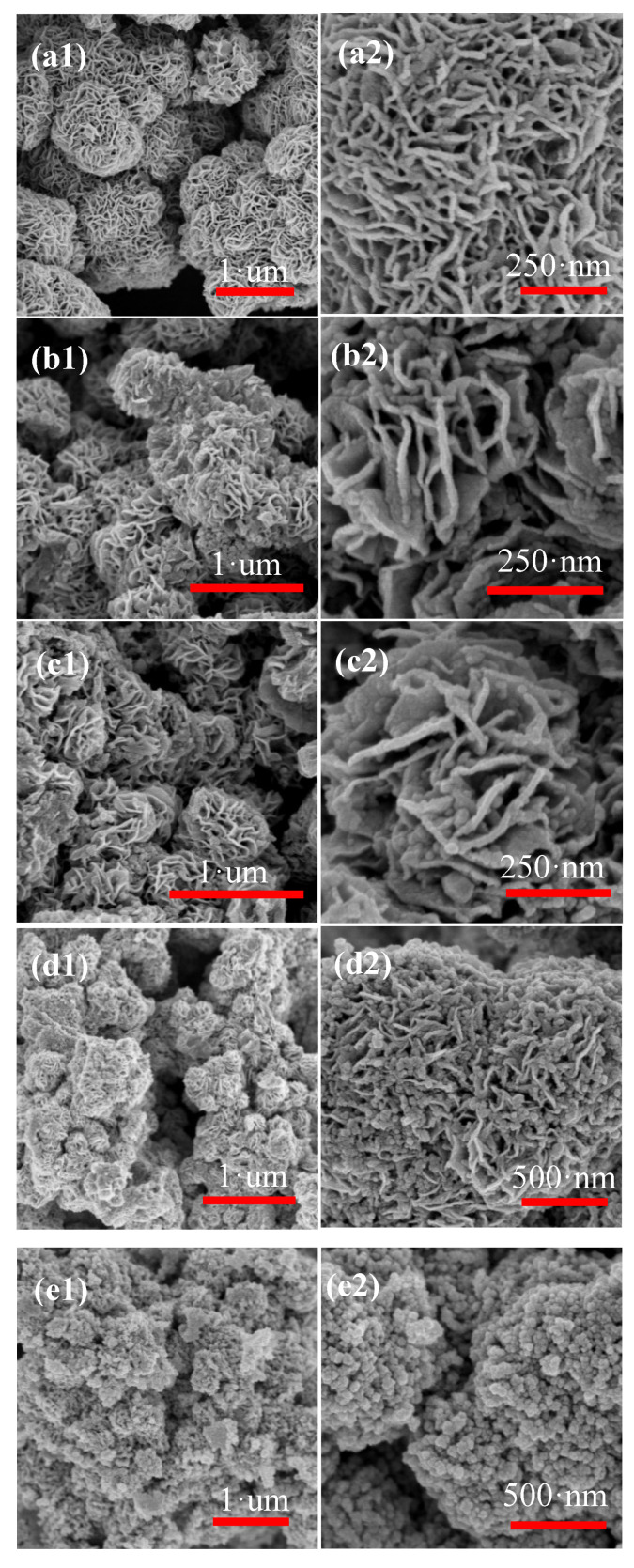
(**a**) Scanning electron microscopy (SEM) of pure MoS_2_, (**b**) sample S1, (**c**) sample S2, (**d**) sample S3, (**e**) sample S4.

Figure 5 shows SEM images of pure MoS_2_ and four different ratios of ZnO-MoS_2_ nanocomposites. It can be seen that pure MoS_2_ are flower-like structures with good uniformity in Figure 5a1. The diameter of a single MoS_2_ crystal formed by numerous sheet-like MoS_2_ with uniform thickness is about 700 nm observed in the high magnification image (Figure 5a2), and there are large porosities among the sheet-like MoS_2_. Figure 5b–e show four different composite ratio ZnO-MoS_2_ nanocomposites, which show that these particle sizes have little change in the range from 0.5 to 1.0 μm with excellent uniformity. In the high resolution, it can be more evidently found the number of ZnO particles increases in the pores and on the outer surfaces of MoS_2_ nanosheets with the addition of Zn for sample S2 and S3. Additionally, for the sample S4, ZnO particles have completely wrapped the MoS_2_, causing the particle shape change from flower-like to stacked spherical particles. Therefore, with the increase in doping concentration, the uniformity is getting better, but too high doping concentration will change the particle shape. Sample S3 with optimal doping concentration was selected for the next characterization test.

**Figure 6:**
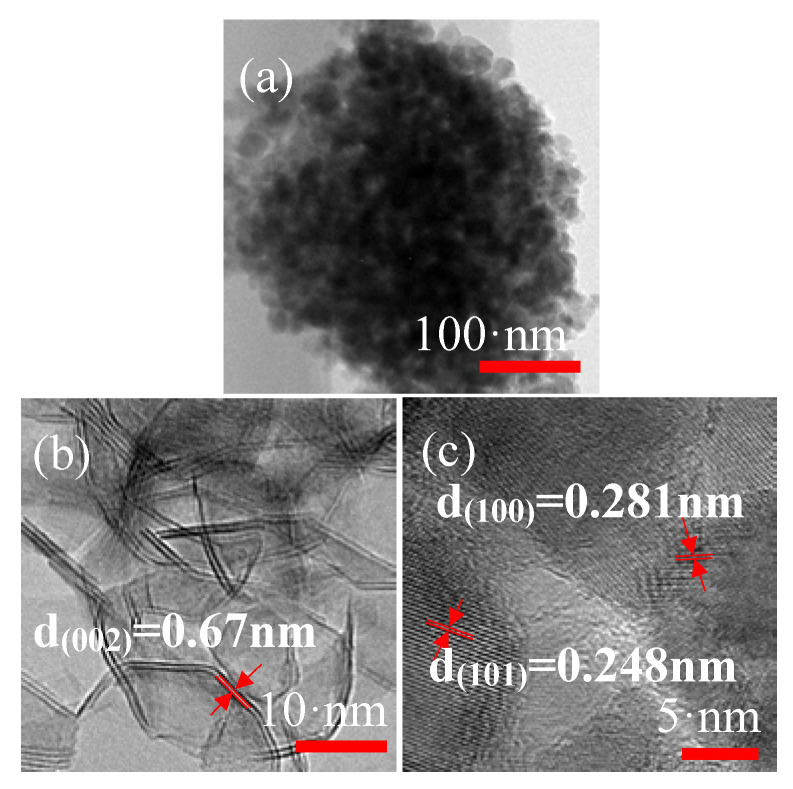
(**a**) Transmission electron microscopy (TEM) of ZnO-MoS_2_ nanocomposite S3. (**b**) HRTEM of MoS_2_ and (**c**) ZnO particles

It can be seen that the TEM patterns of ZnO-MoS_2_ nanocomposites are sphere-like in the central black area with a little light dots in Figure 6a, which means that the internal structure of the composites is not a solid sphere, and there are still pores among MoS_2_ nanosheets. The lighter area around nanocomposites is composed of ZnO wrapped in the MoS_2_ nanosheets, which further proves the good uniformity. The HRTEM analysis of the MoS_2_ nanosheets presents irregular lattice fringes with 0.67 nm (Figure 6b) that corresponds to the MoS_2_ (002) crystal plane. Figure 6c can clearly show regular and orderly lattice fringe spacing that is 0.281 nm corresponds to the ZnO (110) plane and 0.248 nm to the ZnO (101) plane.

**Figure 7:**
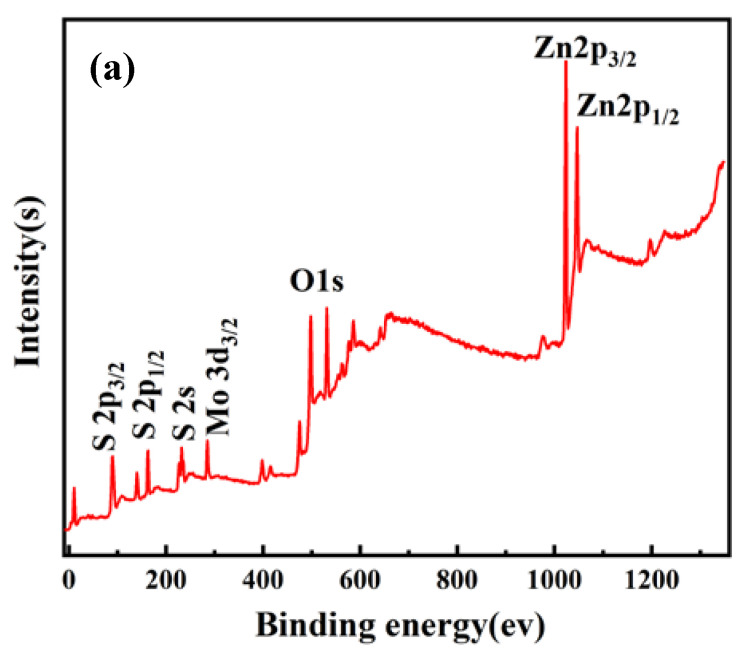

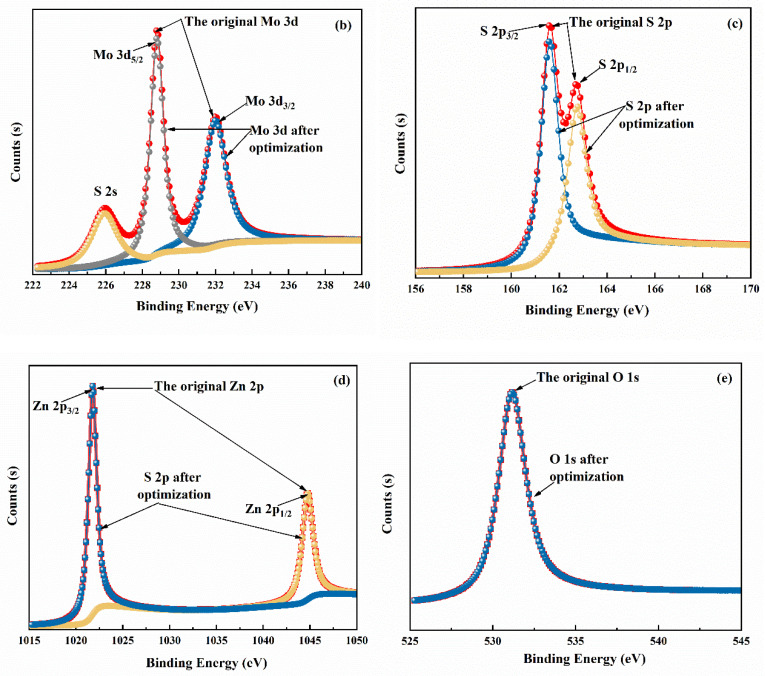
X-ray photoelectron spectroscopy (XPS) data graph in ZnO-MoS_2_ nanocomposite in S3: (**a**) all elements spectrum; (**b**) Mo 3 d spectrum; (**c**) S 2 p spectrum; (**d**) Zn 2 p spectrum; (**e**) O 1 s spectrum.

To characterize the elements and valence in the nanocomposites, the sample S3 is further studied with XPS spectroscopy. As shown in Figure 7a, it can be found that there are three peaks and two of the peaks at 232.3 and 229.1 eV are caused by the binding energy of Mo 3 d3/2 and Mo 3 d5/2, respectively, which proves the existence of Mo^+4^. The peaks appearing in Figure 7b are located at 163.2 and 161.7 eV are attributed to the S 2 p1/2 and S 2 p3/2 of S^−2^, respectively. The two peaks in Figure 7c located at 1045.0 and 1022.7 eV are in agreement with Zn 2 p1/2 and Zn 2 p3/2 of Zn^+2^. Figure 7d is the XPS data of oxygen with the binding energy of 531.9 eV, which is attributed to the O 1 s of O^−2^. Therefore, four characterization methods proved that the ZnO nanoparticles were well doped in the MoS_2_ with little impurities.

### 3.2. Gas-Sensing and Aging Characteristics

**Figure 8:**
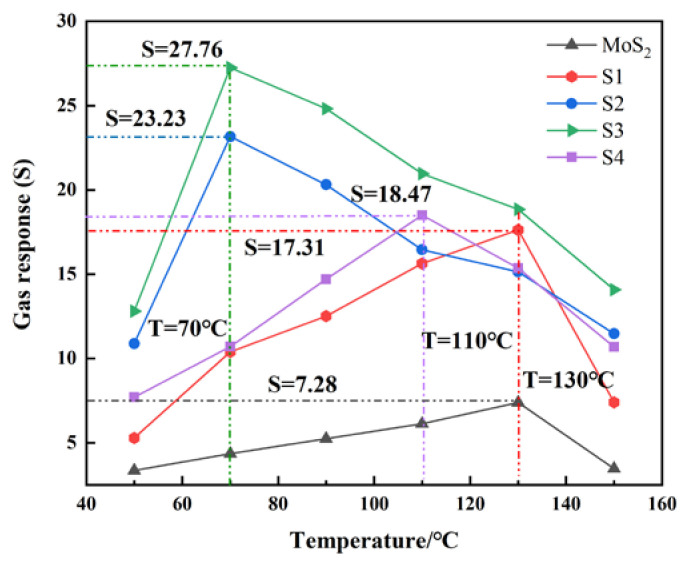
Temperature characteristic curve of gas-sensing material to 15 μL/L C_2_H_2_.

To analyze the influence of the composite structures on the gas-sensing response and find the optimal working temperature of gas sensors, the temperature characteristics of gas-sensing materials are tested. The temperature characteristic curves of each gas-sensing material to 15 μL/L C_2_H_2_ are given in Figure 8. Because MoS_2_ is a narrow-band-gap semiconductor gas-sensing material with low excitation energy, the temperature range is set at 50~150 °C [32,33,34]. It can be seen that the change trend of the gas-sensing response increases first and then decreases accompanied by a rise in working temperature. Among them, the optimal working temperature of pure MoS_2_ and S1 is at 130 °C, and the corresponding gas-sensing response are 7.28 and 17.31, respectively; the optimal working temperature of S2 and S3 is at 70 °C, and the corresponding gas-sensing responses are 23.23 and 27.76, respectively; the optimal working temperature of S4 with the maximum doping ratio is 110 °C, and the corresponding gas-sensing response is 18.47.

The optimal operating temperature gradually decreases with the doping ratio increasing, and the gas-sensing response of S3 is much higher than other nanocomposite materials and pure MoS_2_. The phenomenon is explained by the chemical activation energy being small at a low temperature, and the reaction between C_2_H_2_ and gas-sensing materials are prevented with the existence of a potential barrier. Therefore, with the increase in temperature, the chemical activation energy increases rapidly and the gas-sensing response enhances. As the temperature rises further, oxygen containing functional groups disappears, which causes the gas response to decrease at a low speed. In a word, as a certain amount of compound ratio increases, the optimal working temperature gradually decreases and the gas-sensing response has also been significantly improved compared to the pure MoS_2_.

**Figure 9:**
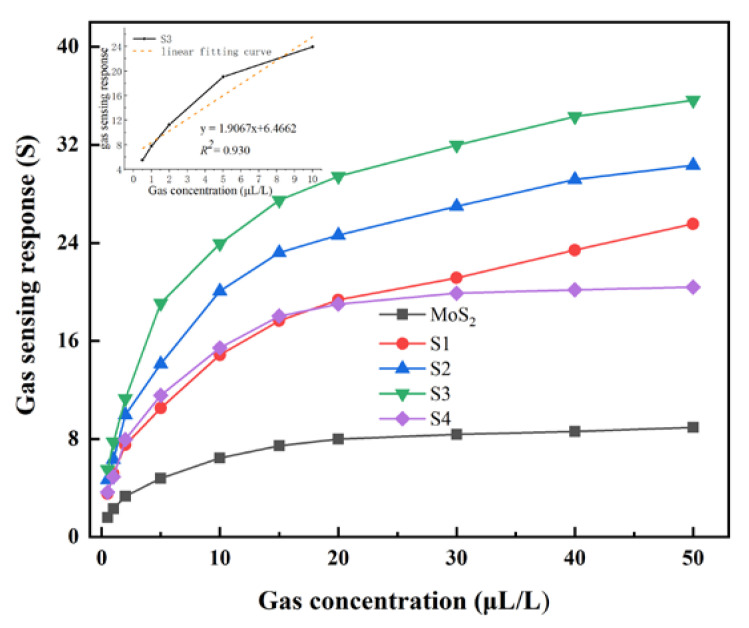
Concentration characteristic curve of gas sensitive material to C_2_H_2_.

The detection range of the C_2_H_2_ gas concentration is selected from 0.5 to 50 μL/L (0.5, 1, 2, 5, 10, 15, 20, 30, 40, 50 μL/L). Figure 9 is the concentration characteristic curve to C_2_H_2_ gas, gas-sensing response gradually increases but the growth rate becomes slower with the concentration increasing, which shows that the concentration of C_2_H_2_ gas is positive feedback to the response of the material. From the Figure 9, it can also be observed that pure MoS_2_ exhibits the worst gas-sensing characteristics. When ZnO particles are combined with MoS_2_, the gas-sensing response value of the material increases significantly. However, the gas-sensing response of S4 showed a relatively large decrease compared with S3. It can be explained that the addition of excessive ZnO particles caused the the pore structure of MoS_2_ disappears. This phenomenon indicates that the proper doping of a certain concentration of ZnO can have a positive effect on the gas properties of MoS_2_. Additionally, in the range of 0.1 to 10 μL/L C_2_H_2_ gas (the inset of Figure 9), the function after the linear fitting of the gas-sensing material is y = 1.9067x + 6.4662, and the corresponding linear correlation coefficient is 0.930, which indicates a low detection limit of the gas sensors with 5.52 gas-sensing response at 0.5 μL/L C_2_H_2_.

**Figure 10:**
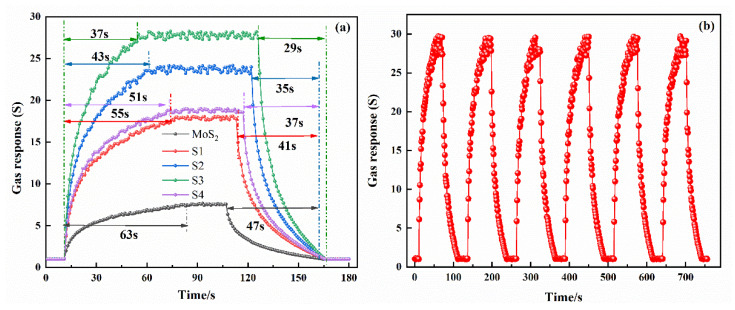
(**a**) Dynamic response–recovery characteristic curve of each gas-sensing material to 15 μL/L C_2_H_2_. (**b**) Repeatability experiment of S3 composite material.

Figure 10a is the dynamic response–recovery characteristic curve of five gas-sensing materials to 15 μL/L C_2_H_2_. It can be found that sample S3 showed the fastest response, with a 37 s response time and a 29 s recovery time. Figure 10b is the dynamic response curve of sample S3 under five cycles. It can be found that the gas-sensing response value of S3 composite material does not obviously change during five continuous test cycles, and the time required for each measurement cycle is also approximately equal. The gas-sensing response of each measurement cycle will return to the base point. This phenomenon shows that the test stability of gas-sensing materials is good in a short time.

**Figure 11:**
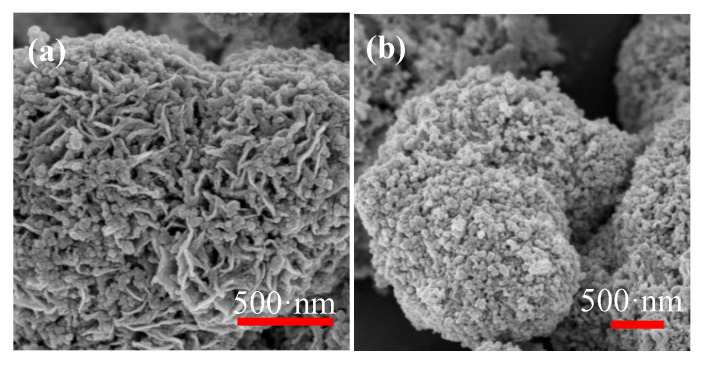
(**a**) SEM of S3 composite material without aging and (**b**) aged for 20 days.

Figure 11 is the scanning electron micrograph comparison of S3 composite material without aging and after 20 days of aging in a dark vacuum environment. Compared with the Figure 11a without aging, it is obviously found that the crystal structure of ZnO-MoS_2_ nanocomposites changes greatly, and the sheet structures of MoS_2_ and the small particles of ZnO appear to grow and agglomerate, which means the porosity of the material decreases and the particle size increases.

**Figure 12:**
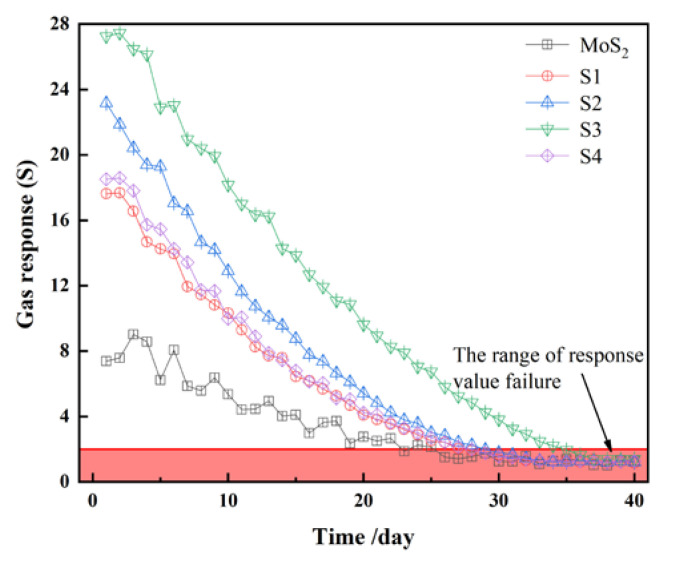
Gas-sensing response curve of each gas-sensing material during the 40-day aging process.

Figure 12 shows the attenuation curve of the gas-sensing response of each material to 15 μL/L C_2_H_2_ during the 40-day aging process. The pure MoS_2_ gas-sensing response value is the smallest. After 24 days of aging, the gas-sensing response drops below 2 and finally jumps between 1 and 1.2. In addition, compared with the composite materials, the decline process of pure MoS_2_ is not smooth, with a large fluctuation and even a slight increase in the response value after aging. It may be that a few crystals are oxidized during the experimental test, which turns the pure MoS_2_ into a MoS_2_-MoO_2_ composite material and causes an increase in the response value. However, the curves of nanocomposites with a certain proportion of heterojunction structure are no jitter.

**Figure 13:**
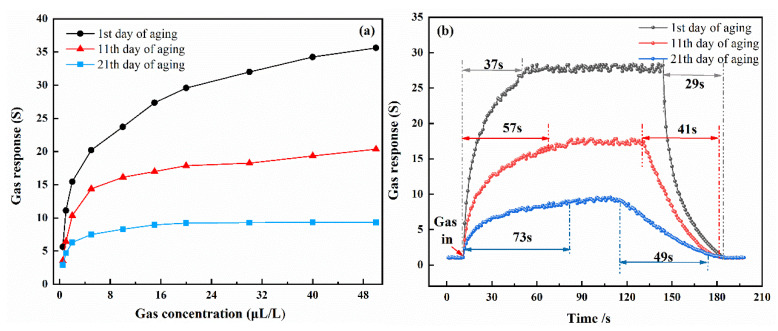
(**a**) Concentration characteristic curves of sample S3 under different aging time. (**b**) Dynamic response–recovery characteristic curve of S3 sample under different aging time.

Concentration characteristic curves of sample S3 under different aging days are depicted in Figure 13a. As the aging time increases, the gas-sensing response value also decreases and the attenuation degree is more obvious under high concentration gas. Figure 13b is the dynamic response curve of sample S3 in 15 μL/L C_2_H_2_ under different aging days. It can be clearly seen that the response and recovery speed of the material after aging has decreased. The specific response and recovery time are shown in the Figure 13b. It means that the material aging not only causes the attenuation of the response value, but also the response and recovery speed of the material is much slower.

### 3.3. Gas-Sensing and Aging Mechanism

The sample S3 shows the best gas-sensing performance in all aspects, including response and recovery time, a low temperature, and so on. Meanwhile, sample S1, sample S2 and sample S4 all present better gas-sensing characteristics, and the optimal working temperature of ZnO-MoS_2_ nanocomposites with four different doping ratios is also lower than the pure MoS_2_. Therefore, a sensing mechanism should be proposed to give an explanation to the above phenomenon.

Through the above experimental results with bad gas-sensing response to C_2_H_2_ gas and the Fermi level close to valence band (EV), it can be drawn that MoS_2_ performed p-type property [35,36]. When MoS_2_ is in the air at ambient temperature, oxygen molecules are absorbed on the surface of MoS_2_ and capture electrons from conduction band (EC) [37,38,39]. The processes of these transformations are as follows (Equations (1)–(4)):

(1) $$O_{2}(\mathrm{gas})\to O_{2}(\mathrm{ads})$$

(2) $$O_{2}(\mathrm{ads})+e^{-}\to O_{2}^{-}(\mathrm{ads})(T<100\;{}^{\circ}C)$$

(3) $$O_{2}^{-}(\mathrm{ads})+e^{-}\to 2O^{-}(\mathrm{ads})(100<T<300\;{}^{\circ}C)$$

(4) $$O^{-}(\mathrm{ads})+e^{-}\to O^{2-}(\mathrm{ads})(T>300\;{}^{\circ}C)$$

When C_2_H_2_ as reducing gas is injected into the sealed vessel, it will react with oxygen ions and release electrons to MoS_2_.The process is presented below in Equations (5)–(7) [40,41]. It will cause resistance decrease because the electrons were liberated, causing the depletion layer width decrease.

(5) $$C_{2}H_{2}(\mathrm{gas})+\frac{5}{2}O_{2}^{-}(\mathrm{ads})\to 2{\mathrm{CO}}_{2}{+H}_{2}O+\frac{5}{2}e^{-}(T<100\;{}^{\circ}C)$$

(6) $$C_{2}H_{2}{(\mathrm{gas})+5O}^{-}(\mathrm{ads})\to 2{\mathrm{CO}}_{2}{+H}_{2}{O+5e}^{-}(100<T<300\;{}^{\circ}C)$$

(7) $$C_{2}H_{2}{(\mathrm{gas})+5O}^{2-}\left(\mathrm{ads}\right)\to 2{\mathrm{CO}}_{2}{+H}_{2}{O+10e}^{-}(T>300\;{}^{\circ}C)$$

**Figure 14:**
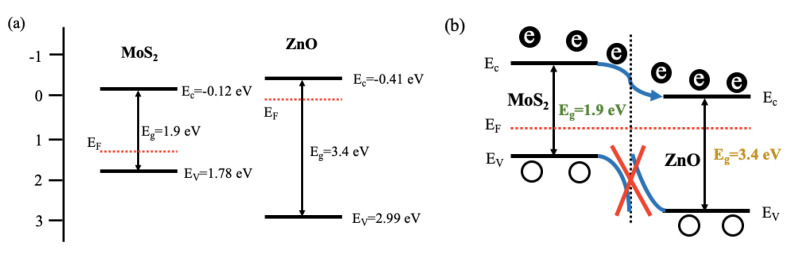
(**a**) Schematic diagram of the energy band positions between MoS_2_ and ZnO before and (**b**) after contacting.

For sample S3 gas sensor with optimal performances, some possible reasons will be tried to give as follows. The main reasons are the positive reaction between two sensing materials and p-n heterojunctions formed between MoS_2_ nanosheets and ZnO nanoparticles. Based on the thermionic conduction mechanism, it can be concluded that electrons with higher energy are easier to move and the holes are prevented migrating from ZnO to MoS_2_ due to the exsistence of potential barrier [42,43,44,45,46]. So, the barrier height and electrons itself energy will be important decisive elements. When the nanocomposites are in the air, oxygen molecules will grasp electrons from the conduction band of ZnO nanoparticles causing the formation of depletion of ZnO with larger width than pure ZnO [47]. Meanwhile, the number of holes of ZnO nanoparticles will increase. All of these changes will form the barrier height (Figure 14).

**Figure 15:**
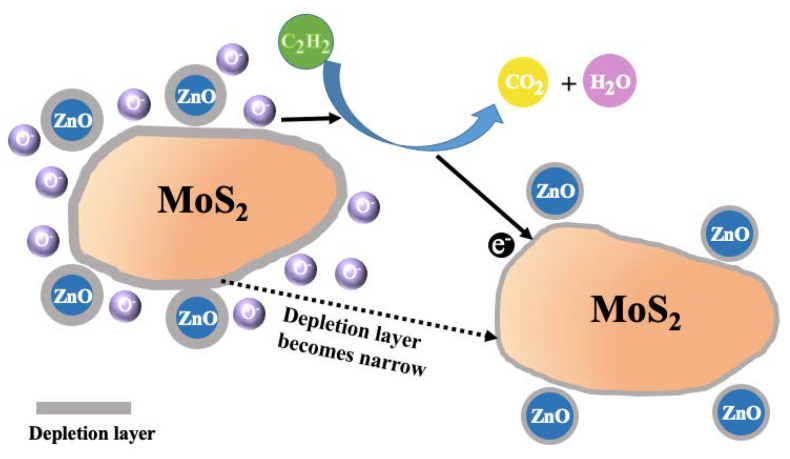
Schematic diagrams of surface sensing reaction of the ZnO-MoS_2_ nanocomposites sensor exposure C_2_H_2_ gas.

C_2_H_2_ will react with oxygen ions and release electrons to the ZnO when nanocomposites are surrounded by C_2_H_2_ gas, as illustrated in Figure 15. This will cause the depletion of ZnO decrease and the barrier height reduce pristine level. Because of the huger change in the width of the depletion layer, the response value increases more obviously than that of pure MoS_2_.

## 4. Conclusions

The ZnO-MoS_2_ nanocomposites were prepared by the two-step hydrothermal method and proved by the consequences of SEM, XRD, TEM and XPS. The acetylene sensing performances of ZnO-MoS_2_ nanocomposites were greatly improved, which exhibited superior repeatability and good long-term stability. Meanwhile, due to the formation of p-n heterojunctions and active interaction, the optimal working temperature of acetylene gas sensors dropped to 70 °C. In addition, ZnO-MoS_2_ nanocomposites increase the thickness of the depletion layer and increase the resistance change rate after reacting with acetylene. The gas-sensing mechanism can draw the conclusion that the ZnO-MoS_2_ nanocomposites can reduce the working temperature, thereby delaying the aging.
